# Metabolic and Immunometabolic Neuroprotection in Glaucoma: Mitochondrial Dysfunction, Neuroinflammation, and Emerging Therapeutic Strategies

**DOI:** 10.3390/molecules31193367

**Published:** 2026-09-22

**Authors:** Alessio Martucci, Chiara Anselmi, Enrico Romano, Lorenzo Bello, Francesco Aiello, Raffaele Mancino, Rossella Russo, Carlo Nucci

**Affiliations:** 1 Ophthalmology Unit, Department of Experimental Medicine, University of Rome “Tor Vergata”, Via Montpellier 1, 00133 Rome, Italy; chiara.anselmi@ptvonline.it (C.A.); lorenzo.bello@ptvonline.it (L.B.);; 2 Department of Sense Organs, Sapienza University of Rome, 00185 Rome, Italy; enrico.romano@uniroma1.it; 3 Department of Pharmacy, Health and Nutritional Sciences, Section of Preclinical and Translational Pharmacology, University of Calabria, 87036 Rende, Italy

**Keywords:** glaucoma, CoenzymeQ10, nicotinamide, pyruvate, citicoline, neuroinflammation, neuroimmune dysregulation

## Abstract

Glaucoma is increasingly recognized as a multifactorial neurodegenerative optic neuropathy in which intraocular pressure (IOP) is one of several stress amplifiers rather than a sufficient etiological explanation. Vascular dysregulation, mitochondrial and metabolic vulnerability, oxidative stress, excitotoxicity, glial dysregulation, and persistent neuroimmune activation can converge on retinal ganglion cell (RGC) and optic nerve injury. This broader framework is particularly relevant to normal-tension glaucoma (NTG), which represents a large proportion of primary open-angle glaucoma in Asian populations, and to patients who continue to progress despite IOP being maintained within the target range. Consequently, mitochondrial dysfunction, metabolic impairment, oxidative stress, and the associated neuroinflammatory response are not merely downstream adjunctive targets: they are core disease mechanisms and potential treatment targets in pressure-independent and pressure-resistant glaucoma. Coenzyme Q10 (CoQ10), nicotinamide, pyruvate, and citicoline are emerging metabolic and neuroprotective agents whose primary actions involve mitochondrial bioenergetics, redox homeostasis, membrane integrity, and cellular stress responses, with potential secondary effects on neuroinflammatory pathways. Chronic activation of microglia and astrocytes promotes the release of pro-inflammatory cytokines, reactive oxygen species, and apoptotic mediators, leading to RGC degeneration. CoQ10 may counteract these mechanisms through antioxidant activity and stabilization of mitochondrial function; nicotinamide, a precursor of NAD^+^, may enhance mitochondrial resilience and suppress inflammatory cascades associated with energy failure; pyruvate supports aerobic metabolism and may limit oxidative, inflammatory, and excitotoxic injury; and citicoline supports membrane homeostasis, phospholipid synthesis, neuronal repair, and visual-pathway function. Combined targeting of these interconnected pathways may enhance RGC resilience independently of, and in addition to, IOP reduction. However, current clinical evidence remains insufficient to establish sustained neuroprotection or disease-modifying efficacy. This review examines the interplay among these mechanisms in glaucomatous neurodegeneration and critically discusses the experimental and clinical evidence supporting metabolic and immunometabolic neuroprotective strategies as potential adjuncts to conventional intraocular pressure-lowering therapy.

## 1. Introduction

Glaucoma is a progressive, multifactorial neurodegenerative optic neuropathy and a leading cause of irreversible blindness worldwide, characterized by the selective loss of retinal ganglion cells (RGCs), optic nerve degeneration, and corresponding visual field (VF) defects [1]. Elevated intraocular pressure (IOP) is the most well-established modifiable risk factor for glaucoma onset and progression, but it should be viewed as one of several stress amplifiers rather than as a universal primary etiological driver. This distinction is clinically important because normal-tension glaucoma (NTG) develops without measured IOP exceeding the statistically normal range, accounts for a substantial proportion of primary open-angle glaucoma and as much as 70–90% in some Asian populations, and can progress despite apparently adequate pressure control. Similarly, a clinically important subset of patients with pressure-lowered glaucoma continues to lose RGCs and visual function after reaching an individualized target IOP [2]. These observations indicate that IOP exposure interacts with, but does not fully account for, vascular dysregulation, impaired ocular perfusion, mitochondrial susceptibility, metabolic failure, oxidative stress, excitotoxicity, and neuroimmune dysregulation [3]. When present, IOP-related mechanical and ischemic stress can disrupt axonal transport and amplify these processes, increasing reactive oxygen species (ROS) and the release of damage-associated molecular patterns (DAMPs) that activate innate immune pathways [4].

Resident glia respond to these stress signals by releasing cytokines, including tumor necrosis factor-α (TNF-α), interleukin-1β (IL-1β), and interleukin-6 (IL-6), which affect RGC somas, axons, and synapses [5,6].

Within this inflammatory milieu, synaptic structures are particularly vulnerable. Pro-inflammatory cytokines, together with complement cascade activation, drive synapse elimination, dendritic remodelling, and early functional disconnection of RGCs from their central targets [5]. This synaptic dysfunction represents a critical initiating step in anterograde trans-synaptic degeneration, through which pathological signals propagate from the retina to second- and third-order neurons in the lateral geniculate nucleus (LGN) [7,8] and visual cortex [3]. At the same time, retrograde signalling mechanisms further contribute to neuronal loss, highlighting the bidirectional nature of degeneration along the visual pathway.

Importantly, neuroinflammation is not confined to the site of initial injury but spreads across synaptically connected regions, where activated microglia and infiltrating immune cells perpetuate cytokine production and sustain chronic inflammation [3]. Additional mechanisms, including activation of intrinsic axonal degeneration pathways such as SARM1 and impaired neurotrophic support, further facilitate the propagation of damage across neuronal networks.

Collectively, these findings support a multifactorial stress-amplification model that links local retinal injury to central visual-pathway degeneration, including when IOP remains within the statistically normal or therapeutically targeted range.

The aim of this review is to critically analyze metabolic and immunometabolic neuroprotective strategies for glaucoma, with particular focus on CoQ10, nicotinamide, pyruvate, and citicoline and on their capacity to target pressure-independent mechanisms relevant to NTG and to disease progression despite adequate IOP lowering.

## 2. Materials and Methods

This narrative review was based on a structured literature search conducted in PubMed/MEDLINE, Scopus, and Web of Science from database inception to July 2026. Google Scholar was additionally used for citation tracking and to identify potentially relevant publications not retrieved through the primary database search. The reference lists of eligible articles and relevant reviews were also manually screened to identify additional studies.

The search strategy combined terms related to glaucoma and retinal ganglion cell degeneration with terms describing neuroinflammatory, metabolic, and neuroprotective mechanisms. The principal keywords included: “glaucoma”, “retinal ganglion cells”, “optic nerve”, “neurodegeneration”, “neuroinflammation”, “microglia”, “astrocytes”, “Müller cells”, “complement system”, “oxidative stress”, “mitochondrial dysfunction”, “metabolic impairment”, “glutamate excitotoxicity”, “neuroprotection”, “Coenzyme Q10”, “ubiquinone”, “ubiquinol”, “nicotinamide”, “NAD^+^”, “pyruvate”, “mitochondrial pyruvate carrier”, “citicoline”, and “CDP-choline”. These terms were combined using the Boolean operators “AND” and “OR”, with search strings adapted to the requirements of each database.

Peer-reviewed articles published in English were considered eligible. Given the translational scope of the review, the selection included clinical trials, observational studies, experimental animal studies, in vitro investigations, systematic and narrative reviews, and relevant consensus or position statements. Studies were included when they addressed: (i) neuroinflammatory and neuroimmune mechanisms involved in glaucomatous neurodegeneration; (ii) mitochondrial dysfunction, oxidative stress, metabolic impairment, or glutamate excitotoxicity affecting retinal ganglion cells; or (iii) the neuroprotective, metabolic, antioxidant, neuromodulatory, or immunomodulatory effects of Coenzyme Q10, nicotinamide, pyruvate, or citicoline.

Studies conducted in neurological or retinal disorders other than glaucoma were retained when they provided mechanistic evidence directly relevant to the pathways discussed, including NF-κB signalling, inflammasome activation, glial reactivity, NAD^+^-dependent regulation, mitochondrial apoptosis, phospholipid metabolism, and glutamate homeostasis. In these cases, the non-glaucomatous experimental context was explicitly acknowledged in the interpretation of the findings.

Duplicate records, non-peer-reviewed publications, conference abstracts without sufficient methodological information, articles not available in English, and studies without a clear relationship to the molecular or cellular mechanisms addressed in this review were excluded. Titles and abstracts were initially screened for relevance, followed by full-text assessment of potentially eligible publications. Study selection was guided by relevance to the review objectives, methodological robustness, recency, and historical or mechanistic importance. Seminal studies published before the main period of recent literature were retained when necessary to describe the development of key concepts or experimental models.

Because this work was designed as a narrative review rather than a systematic review or meta-analysis, no formal protocol was registered, and no quantitative synthesis or standardized risk-of-bias assessment was performed. The evidence was instead critically integrated according to study design, experimental context, translational relevance, and level of clinical validation.

## 3. Neuroinflammation and Neuroimmune Dysregulation in Glaucoma

Neuroinflammation in glaucoma is best understood as a chronic, spatially organized, and self-amplifying response to neuronal and axonal stress rather than as a conventional inflammatory reaction [9,10]. The optic nerve head (ONH) is a major site at which multiple stressors converge: IOP-related biomechanical strain, vascular dysregulation, ischemia, impaired perfusion, mitochondrial dysfunction, and metabolic stress can each compromise axonal transport and RGC axons. Their relative contribution is likely to vary among phenotypes; in NTG and in eyes that progress at target IOP, non-pressure stressors may predominate or maintain injury after pressure-related load has been reduced. Mechanosensitive signalling and intracellular Ca^2+^ overload promote the extracellular release of adenosine triphosphate (ATP), while stressed RGCs, damaged axons, and dysfunctional mitochondria release damage-associated molecular patterns (DAMPs), including heat-shock proteins (HSPs), high-mobility group box 1 (HMGB1), mitochondrial DNA, oxidized proteins, and ROS. These signals are detected by pattern-recognition receptors expressed by microglia, astrocytes, and Müller cells, particularly toll-like receptors (TLRs) and purinergic P2X7 receptors [11,12]. TLR engagement activates MyD88- and TRIF-dependent signalling and the transcription factor NF-κB, whereas ATP-mediated P2X7 activation induces K^+^ efflux, ROS generation, and lysosomal destabilization, thereby promoting assembly of the NLRP3 inflammasome [13]. Activated NLRP3 subsequently stimulates caspase-1-dependent maturation of IL-1β and IL-18, establishing an inflammatory feedback loop that further increases glial activation and RGCs’ vulnerability [14,15].

Microglia are among the earliest cellular responders to glaucomatous stress, and their activation may precede detectable RGC loss [5,14,16,17]. Under physiological conditions, ramified microglia continuously survey the retinal microenvironment, remove cellular debris, regulate synaptic remodelling, and provide trophic support. During the early phases of injury, these functions may remain adaptive and contribute to tissue repair [18]. Persistent biomechanical, oxidative, or metabolic stress, however, induces a transition toward amoeboid, highly phagocytic, and pro-inflammatory states characterized by the release of TNF-α, IL-1β, IL-6, chemokines, nitric oxide, ROS, matrix metalloproteinases, and complement components. The traditional M1/M2 classification is useful as a conceptual model but does not fully represent the functional heterogeneity and stage-dependent plasticity of microglial populations [13,16]. Microglial signals also induce reactive changes in astrocytes and Müller cells, while astrocyte-derived mediators reciprocally regulate microglial migration, complement expression, and phagocytosis [17,19]. Astrocytes can initially preserve tissue architecture, vascular-barrier integrity, metabolic support, and glutamate homeostasis; with prolonged reactivity, however, astrocytes reduce their neurotrophic and antioxidant support, remodel the extracellular matrix, and amplify inflammatory signalling through their extensive gap-junction networks [18,20]. Müller-cell dysfunction additionally impairs extracellular glutamate clearance, contributing to excitotoxicity. In this context, soluble glia-derived TNF-α provides a direct molecular link between neuroinflammation and excitotoxic RGC death by increasing the surface expression of Ca^2+^-permeable AMPA receptors, thereby promoting Ca^2+^ overload, mitochondrial dysfunction, and axonal cytoskeletal damage [21,22].

These interactions can be integrated into a self-sustaining mitochondrial-neuroinflammatory closed loop. Initial impairment of mitochondrial electron transport chain (ETC) function increases electron leakage and mitochondrial ROS production. ROS damage mitochondrial proteins, lipids, and DNA and promote the extracellular release of mitochondrial DNA, oxidized molecules, ATP, and other DAMPs. Recognition of these signals by microglial TLRs, P2X7 receptors, and inflammasome pathways reinforces microglial activation and the secretion of TNF-α and other inflammatory mediators [12,13]. TNF-α-driven NF-κB signalling can, in turn, impair mitochondrial biogenesis and respiratory-chain homeostasis, including reduced expression or function of ETC subunits, thereby worsening electron leakage, ATP failure, and ROS generation. The newly generated ROS and DAMPs re-enter the same pathway, converting an initially trigger-dependent response into a feed-forward cycle of mitochondrial failure and glial activation. Once sufficiently established, this loop may persist after the initiating mechanical or ischemic stress has diminished and may become partly independent of external IOP. It therefore provides a mechanistic explanation for continuing degeneration in NTG and in patients who progress despite achieving target IOP, although the relative contribution of each link and the point at which the loop becomes autonomous remain to be established in human glaucoma (Figure 1).

The complement system represents a central component of this innate neuroimmune response [5,9,16]. Its classical, lectin, and alternative pathways converge on C3 cleavage and may ultimately generate the terminal C5b–9 membrane attack complex (MAC). In glaucoma, the classical pathway appears particularly relevant to early synaptic and dendritic injury. C1q acts as a pattern-recognition molecule that labels stressed synapses, dendrites, and damaged cellular elements for removal. C1q expression is increased in ONH microglia before overt axonal degeneration in experimental glaucoma, while C1q deposition has also been detected within RGC dendrites in experimental models, non-human primates, and human glaucomatous retinas [15,23]. This reactivation of a developmental synaptic-pruning pathway may cause inappropriate microglial elimination of otherwise salvageable RGC synapses [16]. Downstream complement activation generates C3a and C5a, which act as anaphylatoxins and recruit or activate microglia and monocyte-derived cells, whereas C3b promotes opsonization and phagocytic clearance. The broader capacity of complement-regulated microglial phagocytosis to contribute to retinal tissue remodeling is also supported by non-glaucomatous retinal models. In a neonatal hyperoxia model, activated microglia engulfed endothelial cell fragments during retinal vessel regression, while partial microglial depletion attenuated early vascular regression and endothelial apoptosis; transcriptomic and immunohistochemical analyses implicated complement signaling in this phagocytic response. Although this model does not reproduce glaucomatous neurodegeneration, it provides additional mechanistic evidence linking complement activation, microglial phagocytosis, and retinal vascular injury [24]. Formation of C5b–9 may directly injure RGCs through membrane lysis; even at sublytic concentrations, MAC can stimulate intracellular Ca^2+^ signalling and further inflammatory activation. Increased deposition of C1q, C3, C5, and MAC has been reported in experimental and human glaucoma, including C5b–9 deposition on RGCs [25]. Nevertheless, complement activity is not uniformly detrimental: C1q inhibition consistently limits early dendritic and synaptic degeneration in experimental models, whereas the effects of C3 vary according to disease stage, cellular source, and downstream receptor signalling. Early C3 activity may support protective astrocytic responses, while excessive or prolonged C3 activation and C3a receptor signalling can promote microglial recruitment and optic nerve degeneration. These findings argue against indiscriminate complement suppression and instead support component-specific and temporally targeted modulation.

From a broader neuroimmunological perspective, the immune privilege of the retina limits but does not abolish communication with the systemic immune system [5,23]. Reactive microglia and astrocytes upregulate major histocompatibility complex molecules, cytokines, chemokines, adhesion molecules, and antigen-presentation pathways. Concurrent dysfunction of astrocytes and vascular endothelial cells may increase blood–retinal barrier permeability, thereby creating a permissive environment for peripheral immune-cell trafficking. In experimental glaucoma, the CCL2/CCR2 chemokine axis promotes trans-endothelial recruitment of circulating monocytes into the ONH and retina [16,26]. These monocyte-derived macrophages may amplify local inflammation through additional secretion of TNF-α, IL-1β, IL-6, and complement components [15]. However, their involvement appears to be transient and most prominent during early or intermediate stages, whereas chronic disease may be maintained predominantly by resident microglia and macroglia. Human evidence for sustained monocyte infiltration remains inconsistent, partly because infiltrating macrophages can acquire microglia-like markers and because the integrity and disease stage of available human tissue samples vary considerably [16,25].

Adaptive immunity may further sustain neurodegeneration after the initiating ocular stress has subsided [5,10]. Oxidative modifications and HSP release increase the antigen-presenting capacity of reactive glia and may alter the immunogenicity of retinal and optic-nerve proteins. Experimental studies suggest that molecular mimicry between microbial and mammalian HSPs can prime cross-reactive CD4^+^ T lymphocytes, which subsequently infiltrate the retina following IOP elevation and contribute to prolonged RGC degeneration. Changes in circulating autoantibody profiles and an imbalance between pro-inflammatory Th1/Th17 cells and regulatory T cells have also been described in patients with glaucoma, although their causal contribution in humans remains incompletely established [27]. Neuroimmune activation is not necessarily confined to the eye: glial reactivity has been demonstrated along the retrobulbar optic nerve and in central visual structures, including the lateral geniculate nucleus, superior colliculus, and visual cortex [15]. Experimental evidence also suggests retinotopically organized and, in some models, contralateral propagation of inflammatory signals. These findings support the view of glaucoma as a neurodegenerative disorder of the entire visual pathway, while the precise contribution of peripheral immune cells to central visual-pathway inflammation remains unresolved [28].

Evidence from human glaucomatous tissue supports the biological relevance of this inflammatory model [25]. Enucleated eyes from patients with advanced glaucoma show increased IBA1 immunoreactivity, reflecting microglial or macrophage activation, together with marked GFAP-positive astrocytic and Müller-cell gliosis in the retina and ONH. Conversely, late-stage samples do not consistently demonstrate increased CD45^+^ leukocytes or CD163^+^ peripheral macrophages, supporting the possibility that peripheral infiltration is an earlier and transient event, whereas established disease is dominated by persistent resident glial reactivity. Overall, this stage-dependent response [5,15] supports modulation of specific inflammatory pathways while preserving the trophic, phagocytic, and immune-regulatory functions of glial cells, rather than broad immunosuppression [29,30].

## 4. Metabolic-Based Neuroprotective Strategies in Glaucoma

Several agents with neuroprotective properties have been investigated and, in some cases, introduced into clinical practice [31]. These include compounds with antioxidant, anti-inflammatory, anti-apoptotic, and mitochondrial stabilizing effects. Among the most studied and available on the market are citicoline, coenzyme Q10 (CoQ10), pyruvate, nicotinamide, pyrroloquinoline quinone (PQQ), homotaurine, berberine, gamma-aminobutyric acid (GABA), and vitamins. Although not all are approved specifically as neuroprotective treatments for glaucoma, their mechanisms of action and a growing body of clinical evidence support their potential role as complementary therapies in glaucomatous neurodegeneration [32]. Within the closed-loop framework described above, CoQ10, nicotinamide, pyruvate, and citicoline are of particular interest as candidate loop-disrupting interventions because they act at complementary mitochondrial, metabolic, redox, membrane, excitotoxic, and glial nodes rather than exclusively downstream of IOP elevation (Table 1).

### 4.1. Coenzyme Q10

The rationale for investigating CoQ10 in glaucoma lies in the close relationship among mitochondrial failure, oxidative stress, apoptotic signalling, and neuroinflammation [32,33]. By stabilizing mitochondrial function and limiting the oxidative and metabolic stressors that activate glial cells, CoQ10 may influence inflammatory responses without acting as a conventional anti-inflammatory drug [34].

CoQ10, or ubiquinone, is an endogenously synthesized, lipid-soluble molecule concentrated mainly in the inner mitochondrial membrane. Within the electron transport chain, it transfers electrons from complexes I and II to complex III, thereby sustaining adenosine triphosphate (ATP) synthesis and cellular energy homeostasis. CoQ10 cycles between oxidized and reduced states; ubiquinol is its principal antioxidant form. This redox cycling allows the molecule to participate simultaneously in mitochondrial energy production and control of the cellular redox environment [32,33].

These functions have particular relevance for RGCs, which require substantial energy to generate action potentials, maintain ionic gradients, support synaptic activity, and transport material along their axons. The unmyelinated axonal segments in the retina and optic nerve head are densely populated with mitochondria and are therefore highly sensitive to impaired oxidative phosphorylation. By facilitating respiratory-chain electron transfer and limiting electron leakage, CoQ10 may help maintain ATP production while reducing mitochondrial ROS generation and mitigating the metabolic vulnerability of RGCs [34].

Its role is not limited to bioenergetics. CoQ10 also acts as a membrane-associated antioxidant, scavenging ROS, limiting lipid peroxidation, and protecting mitochondrial and nuclear DNA as well as cellular proteins from oxidative modification. It may further strengthen endogenous antioxidant defences. In experimental retinal systems, CoQ10 administration increased expression of nuclear factor erythroid 2-related factor 2 (NRF2), a transcription factor that coordinates antioxidant and cytoprotective responses, and normalized markers of mitochondrial oxidative stress [35].

Some data also suggest a more direct effect on inflammatory gene regulation. In macrophage cell lines, CoQ10 reduced lipopolysaccharide-induced TNF-α production and altered NF-κB-dependent transcription [36]. These observations imply that its immunomodulatory activity is not explained solely by nonspecific ROS scavenging, although the molecular targets through which CoQ10 modifies NF-κB signalling remain incompletely defined.

Support for this possibility also comes from experimental systems outside glaucoma. In PC12 cells exposed to β-amyloid, CoQ10 lowered cyclooxygenase-2 expression and prostaglandin E2 production, limited IκB degradation, and prevented nuclear translocation of NF-κB [37]. The result is consistent with interruption of NF-κB-dependent inflammatory signalling in an Alzheimer-type injury model, although the cellular system does not reproduce the in vivo interplay among neurons, microglia, and astrocytes.

An immunomodulatory effect has likewise been observed clinically outside the ophthalmic setting. In a randomized, double-blind trial of relapsing-remitting multiple sclerosis, 12 weeks of CoQ10 supplementation at 500 mg/day reduced circulating TNF-α, IL-6, and matrix metalloproteinase-9 [38]. Although systemic biomarker changes do not establish suppression of inflammation within the central nervous system, they indicate that CoQ10 can influence inflammatory mediators in an immune-mediated neurodegenerative disease.

CoQ10 may also protect RGCs by acting on the mitochondrial apoptotic pathway. Loss of mitochondrial membrane potential and opening of the mitochondrial permeability transition pore can promote cytochrome-c release and caspase-dependent cell death. Ubiquinol supplementation reduced active Bax expression, preserved Bcl-xL and phosphorylated Bad, and inhibited caspase-3 cleavage. Because cytokine signalling and mitochondrial ROS converge on many of the same apoptotic pathways, control of mitochondrial-mediated cell death may represent another point at which the antioxidant, immunomodulatory, and neuroprotective actions of CoQ10 intersect [39].

Excitotoxicity provides an additional link between mitochondrial dysfunction and RGC death [40,41]. In a rat model of transient retinal ischemia induced by acute elevation of IOP, synaptic glutamate increased during reperfusion and preceded delayed apoptosis in the RGC layer [42]; intraocular CoQ10 attenuated both the glutamate rise and retinal injury [43]. A subsequent mechanistic analysis of the same experimental framework showed that abnormal extracellular glutamate and activation of NMDA and non-NMDA receptors contribute to RGC death [41,42], supporting the view that CoQ10-mediated preservation of mitochondrial function and control of oxidative stress may also limit secondary excitotoxicity [40]. Although these findings derive from acute ischemia–reperfusion rather than chronic glaucoma, they identify glutamate dysregulation as a further pathway through which mitochondrial support may protect RGCs [44].

Experimental retinal models provide direct evidence of this broader protective profile. Exposure of porcine retinal organ culture to hydrogen peroxide caused RGC loss, increased microglial cell numbers, and altered oxidative and inflammatory markers. CoQ10 reduced microglial proliferation and the expression of oxidative and inflammatory markers while preserving RGC survival; it also increased NRF2 expression and normalized the mitochondrial stress marker SOD2. The model therefore links antioxidant protection to attenuation of retinal microglial responses, although it reflects acute oxidative injury rather than chronic glaucoma [35].

A mouse model of retinal ischemia–reperfusion produced a similar pattern. Following transient elevation of intraocular pressure, dietary ubiquinol improved RGC survival and reduced astroglial and microglial activation, as shown by lower GFAP and Iba1 responses. It also modified the Bax/Bad/Bcl-xL pathway and prevented caspase-3 cleavage [39]. In this setting, CoQ10 influenced oxidative stress, glial reactivity, and mitochondrial apoptosis within the same injury response.

In ocular hypertension, the most relevant evidence comes from a combined formulation. Treatment with CoQ10 and citicoline prevented the loss of Brn3a-positive RGCs and preserved retinal neuronal integrity [45]. A later analysis of the same therapeutic approach found reduced macroglial and microglial activation in the retina and in central visual structures, including the dorsolateral geniculate nucleus, superior colliculus, and visual cortex [46].

The combination treatment reduced GFAP immunoreactivity and several indices of microglial activation, including changes in soma size, dendritic arbor area, cell number, and P2RY12 expression [46]. These observations suggest that immunometabolic protection may extend beyond the retina to higher visual centres involved in glaucomatous neurodegeneration. Their interpretation nevertheless requires caution: the effects of citicoline and CoQ10 are analysed together, and the studies did not include separate monotherapy groups. The preservation of RGCs and reduction in glial activation therefore cannot be assigned specifically to CoQ10 and may instead reflect additive or synergistic effects [45,46].

Across these models, CoQ10-containing treatments affect several interconnected features of retinal injury, including mitochondrial dysfunction, oxidative stress, glial activation, and apoptosis. The relative importance of each mechanism is likely to vary with the experimental setting, and direct inhibition of defined inflammatory pathways in chronic glaucoma remains less well characterized than the antioxidant and anti-apoptotic effects. Translation is further complicated by pharmacokinetics. CoQ10 is highly lipophilic, has limited gastrointestinal absorption, and shows marked formulation-dependent bioavailability; lipid-based, micellar, nanoparticulate, and prolonged-release preparations have consequently been developed to improve absorption and stabilize plasma exposure [33,47].

In healthy volunteers, a timed-release oral formulation maintained plasma CoQ10 concentrations during repeated administration, and divided dosing produced greater systemic exposure than the same total dose given once daily. The study, however, addressed plasma pharmacokinetics rather than retinal delivery, ocular neuroinflammation, or clinical efficacy in glaucoma [47].

Topical administration overcomes some limitations of oral absorption, but delivery to the retina and optic nerve depends on the excipients and delivery system used. Findings obtained with one preparation should therefore not be generalized automatically to all topical CoQ10 products [32,33].

Clinical evidence in glaucoma remains preliminary. In patients with primary open-angle glaucoma receiving standard IOP-lowering therapy, topical CoQ10 combined with vitamin E was associated with more favourable pattern visual-evoked-potential responses and less deterioration of the RGC layer and retinal nerve fibre layer (RNFL) measurements. These outcomes are compatible with a functional and structural neuroprotective effect, but the contribution of CoQ10 cannot be separated from that of vitamin E. Moreover, visual evoked potentials, VF, and optical coherence tomography (OCT) provide indirect measures of neuronal function and structure and do not demonstrate suppression of retinal or central neuroinflammation [48].

Short-term studies generally report good tolerability of CoQ10 formulations, with no relevant adverse events in the timed-release bioavailability study. Even so, the optimal dose, treatment duration, formulation, ocular penetration, and long-term safety profile for glaucoma have not been established, and evidence for a definitive effect on VF or structural progression remains limited [47].

Accordingly, the anti-inflammatory efficacy of CoQ10 monotherapy in human glaucoma has not been established [33,48]. Larger controlled trials are needed to determine whether it provides sustained neuroprotection and whether any benefit is independent of IOP reduction and concomitant antioxidant or neuroprotective agents [32,45,46].

### 4.2. Pyruvate and Nicotinamide

Among the metabolic approaches under investigation, pyruvate and nicotinamide address two closely related vulnerabilities: inadequate mitochondrial substrate supply and loss of nicotinamide adenine dinucleotide (NAD^+^) homeostasis. Their proposed value in glaucoma is therefore rooted less in direct anti-inflammatory activity than in restoration of the metabolic conditions required for RGC survival. By correcting energy failure and oxidative imbalance, the two compounds may also reduce the signals that drive glial activation and sustain neuroinflammation [32,49].

Pyruvate sits at the interface between cytosolic glycolysis and mitochondrial oxidation. It reaches the mitochondrial matrix through the mitochondrial pyruvate carrier (MPC), which is formed by the essential MPC1 and MPC2 subunits [50,51]. Once transported across the inner mitochondrial membrane, pyruvate is converted to acetyl-CoA by the pyruvate dehydrogenase complex and enters the tricarboxylic acid cycle, supporting oxidative phosphorylation and ATP generation. The severe energetic consequences of pathogenic MPC2 variants illustrate the importance of this pathway for cellular metabolism [52].

Pyruvate also provides protection beyond its role as a fuel. It can directly scavenge hydrogen peroxide and other ROS, thereby limiting damage to proteins, membrane lipids, and mitochondrial and nuclear DNA. Oxidative DNA injury activates poly adenosine diphosphate (ADP)-ribose polymerase-1 (PARP-1), which consumes NAD^+^, impairs NAD^+^-dependent glycolysis, and contributes to metabolic collapse. Exogenous pyruvate may partly circumvent this bottleneck because it can enter mitochondria directly and support oxidative metabolism without first being generated through glycolysis. It may also limit PARP-1 overactivation, preserve intracellular NAD^+^, increase glycogen reserves, and improve neuronal tolerance to ischemic and hypoglycaemic stress [32,53].

A separate mechanism involves glutamate handling. In the circulation, pyruvate serves as a substrate for glutamate-pyruvate transaminase, favouring conversion of glutamate and pyruvate into alanine and α-ketoglutarate. Lower circulating glutamate may promote its clearance from neural tissue and reduce extracellular accumulation, calcium overload, and neuronal hyperexcitability. This mechanism has been explored in cerebral ischemia, traumatic brain injury, epilepsy, and metabolic or β-amyloid-related neuronal damage [53].

The effects of pyruvate metabolism on inflammation are context-dependent. Activated glia and immune cells undergo marked metabolic reprogramming, and the direction of the response can depend on mitochondrial pyruvate flux. In a model of lipopolysaccharide-induced fever and hypothalamic inflammation, MPC inhibition reduced mitochondrial hydrogen peroxide production, NF-κB and NF-IL6 activation, and release of TNF-α and IL-6 [51]. In other pathological contexts, however, exogenous pyruvate lowered ROS accumulation, PARP-1 activation, inflammatory signalling, and neuronal injury [51,53]. These apparently divergent observations suggest that pyruvate’s effect on neuroinflammation is determined by cell type, substrate availability, and the stage and nature of the inflammatory process.

Nicotinamide targets the other side of this metabolic axis. As the amide form of vitamin B3, it contributes to the synthesis of NAD^+^, a cofactor required for glycolysis, the tricarboxylic acid cycle, oxidative phosphorylation, DNA repair, antioxidant defence, calcium signalling, and mitochondrial quality control. Through the NAD^+^ salvage pathway, nicotinamide is converted by nicotinamide phosphoribosyltransferase to nicotinamide mononucleotide and then by nicotinamide mononucleotide adenylyltransferases to NAD^+^. Nicotinamide riboside enters a related but distinct route through NRK1 and NRK2 before generating nicotinamide mononucleotide. The two precursors can therefore increase NAD^+^ availability but should not be regarded as metabolically interchangeable [32,49].

Loss of NAD^+^ provides a direct link between metabolic stress and inflammation. PARP consumes NAD^+^ during the response to oxidative DNA damage, whereas inflammatory activation can accelerate its degradation through CD38. As NAD^+^ levels fall, mitochondrial respiration and NAD^+^-dependent sirtuin activity decline. Restoring the cofactor supports mitochondrial stress responses, FOXO signalling, mitophagy, antioxidant defence, and protein deacetylation [54]. By limiting the accumulation of dysfunctional mitochondria, these effects may also reduce the release of ROS, mitochondrial DNA, and other damage-associated inflammatory signals.

Sirtuin-dependent regulation provides a plausible route from NAD^+^ metabolism to inflammatory transcription. Deacetylation of the p65 subunit of NF-κB can reduce its transcriptional activity and limit expression of cytokines and inflammatory enzymes. In non-glaucomatous models, nicotinamide riboside increased brain NAD^+^ and reduced lipopolysaccharide-induced microglial and astrocytic activation, NF-κB signalling, cytokine production, and secondary neuronal injury [55]. Nicotinamide likewise reduced IBA1-positive microglial activation, TNF-α expression, and acetylated nuclear NF-κB p65 in a murine model of diabetes-associated brain inflammation [56]. These findings cannot be transferred directly to glaucoma, but they support the biological plausibility of an anti-inflammatory effect mediated through NAD^+^ restoration.

Evidence from glaucoma models indicates that these metabolic abnormalities arise early. In DBA/2J mice, ocular hypertensive stress produced progressive retinal pyruvate depletion, glucose accumulation, and transcriptional changes affecting glycolysis, NAD^+^ metabolism, and MPC1/MPC2 expression [57]. The increase in MPC1 and MPC2 may represent an inadequate compensatory attempt to improve mitochondrial pyruvate utilization in the face of declining substrate availability.

Restoring that substrate had a marked protective effect. Oral pyruvate supplementation normalized retinal pyruvate levels and protected RGC somas and axons in both mouse and rat models of glaucoma [57]. In rats, treatment also reduced ED1-positive microglial activation, suggesting that metabolic rescue may be accompanied by attenuation of local inflammation. Whether pyruvate acts directly on NF-κB, NLRP3, or other defined inflammatory pathways in glaucomatous tissue remains unresolved.

These findings may reflect an indirect effect of substrate replenishment on the mitochondrial–glial feedback loop, rather than direct suppression of inflammatory pathways [49,58].

Metabolomic and genetic data further suggest that pyruvate availability may influence glaucoma susceptibility. Higher circulating concentrations of pyruvate, lactate, and citrate were associated with a lower prevalence of glaucoma in individuals with high polygenic risk, while dietary pyruvate reduced IOP elevation and optic nerve injury in mice [59]. These observations are consistent with improved metabolic resilience, although the human association is observational and cannot establish causality.

Nicotinamide has shown similarly strong effects in experimental glaucoma. Retinal NAD^+^ availability declines with age, potentially lowering the threshold at which RGCs become vulnerable to metabolic and pressure-related stress. In DBA/2J mice, oral nicotinamide preserved mitochondrial function and prevented molecular, functional, and structural features of glaucoma; at the highest dose, approximately 93% of treated eyes had no detectable RGC soma or axonal degeneration [60].

In this model, metabolic neuroprotection was demonstrated, whereas microglial and astrocytic responses were not specifically characterized.

Pyruvate and nicotinamide are therefore mechanistically complementary. Nicotinamide replenishes NAD^+^ and supports glycolysis, mitochondrial redox reactions, DNA repair, and cellular stress responses, whereas pyruvate supplies mitochondria directly, scavenges ROS, and may limit PARP-1-mediated NAD^+^ consumption. Their combined use therefore links substrate replacement with NAD^+^ restoration [32,49].

This combination has already been examined in treated open-angle glaucoma. In a phase II randomized, double-masked, placebo-controlled trial, nicotinamide was escalated from 1000 to 3000 mg/day and pyruvate from 1500 to 3000 mg/day. Compared with placebo, treated participants had a greater number of VF locations improving beyond expected test-retest variability. No serious adverse events occurred during the short follow-up, but the trial was not designed to establish long-term prevention of structural or functional progression. The result is therefore better interpreted as short-term neuroenhancement than as definitive disease-modifying neuroprotection [61].

A subsequent phase III randomized trial was designed to test sustained efficacy with nicotinamide 3 g/day and calcium pyruvate 1 g/day in patients with treated open-angle glaucoma. Its outcomes combine VF testing with spectral-domain OCT over longer follow-up [62].

Clinical use is also constrained by safety considerations. Long-term supplementation requires careful safety evaluation, because favorable short-term tolerability does not establish safety during prolonged treatment. This is particularly relevant to high-dose nicotinamide, for which hepatotoxicity, including serious drug-induced liver injury at 3 g/day, has been reported. The joint American Glaucoma Society–American Academy of Ophthalmology position statement advises against doses ≥ 3 g/day outside closely monitored clinical trials. Even when lower doses are considered, collaboration with the patient’s primary care physician and periodic liver-function testing are recommended, while patients with current or previous liver disease should not receive nicotinamide supplementation for glaucoma neuroprotection. Nicotinic acid should not be substituted for nicotinamide, as these compounds are not interchangeable. Further longitudinal studies are needed to establish the optimal dose, treatment duration, and long-term safety of nicotinamide, both alone and in combination with other metabolic agents [63].

Viewed together, pyruvate and nicotinamide are best understood as complementary immunometabolic interventions rather than direct anti-inflammatory drugs [32,49]. However, direct evidence for suppression of specific inflammatory pathways in human glaucoma remains limited, and long-term clinical data are required before nicotinamide and pyruvate can be considered established neuroprotective therapies or disease-modifying treatments for glaucoma [61,62,63].

### 4.3. Citicoline

Citicoline offers a distinct route to neuroprotection in glaucoma, centred on the maintenance and repair of neuronal membranes [64,65]. Through its effects on phospholipid metabolism, mitochondrial membrane integrity, glutamatergic signalling, oxidative stress, and apoptosis, it may preserve RGC function while also limiting the cellular damage that fuels neuroimmune activation [66,67]. Its potential anti-inflammatory influence is therefore closely tied to membrane stabilization and restoration of neuronal homeostasis rather than to classical immunosuppression [68,69].

Chemically, citicoline is cytidine-5′-diphosphocholine (CDP-choline), an endogenous intermediate in the synthesis of phosphatidylcholine [70,71], one of the major phospholipids in neuronal membranes [64]. Pharmaceutical-grade citicoline is identical to the endogenous metabolite. After administration, it is rapidly hydrolysed to cytidine and choline; in humans, cytidine is converted predominantly to uridine, and the circulating metabolites cross the blood–brain barrier before being reused for synthesis of CDP-choline and membrane phospholipids in the central nervous system [71,72].

This biochemical role gives citicoline effects that extend well beyond passive incorporation into membranes. By increasing substrate availability for the Kennedy pathway [70,71], it supports phosphatidylcholine synthesis, membrane repair, synaptic-vesicle formation, and maintenance of neuronal and mitochondrial membrane architecture [72,73]. Such support may be especially relevant to RGCs, whose long axons, high energy demand, and dependence on efficient axonal transport make them vulnerable to combined membrane and mitochondrial injury [64]. Restored phospholipid metabolism may help preserve membrane fluidity, receptor and transporter function, mitochondrial bioenergetics, and synaptic transmission [73,74].

Membrane preservation also explains part of the connection between citicoline and neuroinflammation. Oxidative or ischemic injury activates phospholipases and promotes hydrolysis of membrane phospholipids, releasing arachidonic acid and other free fatty acids. Their subsequent metabolism generates lipid peroxides, reactive aldehydes, prostaglandins, and additional mediators that amplify oxidative and inflammatory damage. Citicoline attenuates the pathological increase in phospholipase A2 activity [75,76], preserves phosphatidylcholine and cardiolipin, and reduces arachidonic acid release and lipid peroxidation [71,77].

This effect should not be confused with direct enzymatic inhibition. Citicoline and its metabolites do not inhibit phospholipase A2 directly in vitro; instead, they appear to limit the injury-induced increase in enzyme activity by maintaining membrane homeostasis and attenuating upstream cellular damage [75,76,77]. Citicoline may also act on glutamate handling, a potential target within the excitotoxic pathway.

In experimental cerebral ischemia, citicoline reduced glutamate release, preserved ATP, increased glutamate uptake, and enhanced membrane expression of the astrocytic transporter EAAT2 [78]. By improving glutamate clearance rather than broadly suppressing physiological neurotransmission, citicoline may interrupt the reciprocal amplification of excitotoxicity, mitochondrial injury, and glial activation [66,76]. These data derive from cerebral ischemia [78], but glutamate-related stress is also relevant to RGC vulnerability in glaucoma [64,73].

Mitochondrial membrane protection adds another layer to this response. Preservation of phospholipids such as cardiolipin can stabilize the organelle, support respiratory-chain function, and limit cytochrome-c release [71,76]. Citicoline has also been associated with lower production of hydroxyl radicals, lipid-peroxidation products, and other ROS [75,77]. This membrane-directed action may also reduce upstream inflammatory signals [66].

Broader immunomodulatory effects have been reported in neurological models unrelated to glaucoma [66,79]. Citicoline reduced NF-κB activation, pro-inflammatory cytokines including TNF-α, IL-1β, and IL-6, oxidative stress, and microglial responses in experimental ischemic stroke, traumatic neural injury, and other neuroinflammatory conditions. However, these actions are context-dependent, and much of the evidence comes from experimental models or reviews rather than direct analysis of glaucomatous retinal tissue [75].

Sirtuin 1 (SIRT1) may provide an additional molecular link. This NAD^+^-dependent deacetylase coordinates mitochondrial homeostasis, oxidative-stress responses, apoptosis, and inflammatory transcription [80,81]. Deacetylation of the RelA/p65 subunit of NF-κB and modulation of p53 can reduce expression of inflammatory mediators and pro-apoptotic genes. SIRT1 may also affect NLRP3 inflammasome activity, although the direction and magnitude of this effect vary with cell type and disease context [82].

In experimental cerebral ischemia, citicoline increased SIRT1 expression in parallel with neuroprotection, whereas pharmacological inhibition or genetic reduction of SIRT1 weakened part of the protective response [83]. SIRT1 upregulation may therefore contribute to the control of inflammation, oxidative injury, and apoptosis [80,81]. This pathway has not, however, been demonstrated directly in human glaucoma or established as the dominant mechanism underlying citicoline’s effects on RGCs [82].

Citicoline also has a distinct neuromodulatory profile [71,76]. In experimental models, citicoline administration has been associated with increased retinal dopamine availability [73,74], while studies in other neurological systems indicate effects on the synthesis and release of acetylcholine, dopamine and additional monoamines [72].

Because dopamine contributes to retinal adaptation, contrast processing, synaptic transmission, and neuronal plasticity [84], this action may help explain some of the functional responses observed during citicoline treatment; it should nevertheless be distinguished from direct anti-inflammatory activity. Experimental studies also support anti-apoptotic and membrane-preserving effects in RGCs. In retinal cultures exposed to glutamate, high glucose, or other metabolic stressors, citicoline reduced TUNEL-positive cells and activation of caspase-3 and caspase-9 while promoting preservation or regrowth of neurites [73,74].

In animal models of optic nerve crush and chemically induced retinal damage, citicoline preserved RGC density, supported Bcl-2 signalling, reduced retinal-layer injury, and maintained markers of cholinergic and dopaminergic neurons [74]. Topical treatment has also been associated with preservation of inner retinal layers in experimental diabetic retinal injury [64,65]. Taken together, these models suggest that citicoline can increase RGCs’ resistance to axonal, excitotoxic, and metabolic stress.

Evidence for a restorative action comes mainly from outside the field of glaucoma. In experimental stroke, citicoline enhanced neurogenesis, synaptic remodelling, and structural recovery during the post-ischemic phase [85]. This raises the possibility that citicoline supports neuronal plasticity as well as cell survival. True regeneration of chronically damaged RGC axons has not been established, however, and the term “neuroregeneration” should be used cautiously in glaucoma.

The non-glaucomatous findings described above [66,79] do not establish a direct effect of citicoline on retinal glia in glaucoma.

Clinical biomarker data from ischemic stroke provide only indirect support. In patients with ischemic stroke and atrial fibrillation, adjunctive citicoline was associated with lower circulating GFAP, neurofilament light chain, and myelin basic protein, findings compatible with reduced reactive astrogliosis, axonal injury, and demyelination. Circulating Iba1 did not fall significantly, and the results cannot be extrapolated directly to retinal neuroinflammation [86].

In this study, the absence of groups receiving the individual compounds prevents these effects from being assigned specifically to citicoline. The findings therefore support investigation of the combination, without establishing the contribution of either compound or demonstrating synergy [46,87].

The clinical literature on citicoline has focused mainly on electrophysiological function. In patients with open-angle glaucoma already receiving hypotensive therapy, intramuscular citicoline improved pattern electroretinogram measures and visual evoked potentials, suggesting greater retinal bioelectrical activity and faster conduction along post-retinal visual pathways [88,89].

During eight years of intermittent treatment, PERG and VEP responses repeatedly improved or stabilized. Partial deterioration during washout periods indicates, however, that at least some of the response was treatment-dependent and may represent reversible neuroenhancement of dysfunctional but viable RGCs rather than permanent restoration of lost neurons [89].

These electrophysiological changes are compatible with citicoline’s membrane-supporting [70,73] and neuromodulatory actions [64,74], as described above [65], but do not show that retinal neuroinflammation has been reduced. Demonstrating such an effect in patients would require inflammatory or glial biomarkers, molecular imaging, or other direct measures of immune activation.

Citicoline has been administered orally, intramuscularly, and topically [64]. Its oral metabolites have high systemic availability, while experimental and preliminary clinical work suggests that topical formulations can reach posterior ocular tissues and modify retinal electrophysiological responses [71,72]. Exposure of the retina, dosage, pharmacokinetics, and treatment duration nevertheless vary across routes and formulations, so results from one preparation should not be generalized to all citicoline products.

The available clinical evidence suggests potential short-term functional benefits but remains insufficient to establish sustained neuroprotection. It is constrained by small study populations, heterogeneous and often intermittent treatment schedules, reliance on electrophysiological or other surrogate endpoints, and the absence of neuroinflammatory biomarkers [64,65]. Improvement in PERG, VEP, or VF measures [88,89] does not by itself establish long-term preservation of RGC structure or axons [67].

This distinction is central to interpretation of the literature. Recovery of function in impaired but viable RGCs is best described as neuroenhancement [88,89]; slowing subsequent cell loss would demonstrate neuroprotection [64,67]; restoration of neurons or axons already lost would be required for neuroregeneration [74,85]. Current evidence most strongly supports a potential neuroenhancing effect and a biologically plausible neuroprotective action, whereas sustained disease modification and true regeneration remain unproven [65].

Taken together, these mechanisms support a membrane-directed neuroprotective and neuromodulatory profile for citicoline [64,73], with possible secondary immunomodulatory effects [66,79].

The anti-inflammatory component of this profile has been studied far more extensively in cerebral ischemia and other neurological disorders than in glaucoma [66,68]. Direct suppression of retinal neuroinflammation by citicoline monotherapy has not been demonstrated in patients [64,69]. Future controlled studies should establish whether treatment provides durable structural and functional protection [67,87], define the most effective formulation and schedule, and determine whether combination with mitochondrial or metabolic agents produces additive or synergistic benefit [46].

**Table 1:** Preclinical and clinical evidence for metabolic interventions in glaucoma and major translational bottlenecks. POAG: primary open angle glaucoma; CoQ10: Coenzyme Q10; IOP: intraocular pressure; TPGS: D-α-tocopheryl polyethylene glycol 1000 succinate; RGCs: retinal ganglion cells; NRF2: Nuclear factor erythroid 2–related factor 2; SOD: Superoxide Dismutase; VEP: Visual evoked potential; RNFL: retinal nerve fiber layer; ED1: Ectodysplasin-1; OAG: open angle glaucoma; IM: intramuscular; PERG: pattern electroretinogram; NAD: Nicotinamide Adenine Dinucleotide; VF: Visual field; OCT: optical coherence tomography; GFAP: Glial Fibrillary Acidic Protein; P2RY12: Purinergic Receptor P2Y12.

Compound/Combination	Key Preclinical Evidence/Model	Key Clinical Evidence	Dose/Regimen	Route	Main Outcomes	Level of Evidence	Major Translational Bottlenecks
CoQ10	Porcine retinal organ culture exposed to H_2_O_2_ [35]; mouse retinal ischemia–reperfusion [39].	POAG patients receiving topical CoQ10 + vitamin E alongside IOP-lowering therapy [48].	Ex vivo: 700 μM, 48 h. Mouse: 1% ubiquinol diet (~1600–2000 mg/kg/day), starting 1 month before ischemia. Clinical: 0.1% CoQ10 + 0.5% vitamin E TPGS, twice daily for 12 months.	Culture medium; oral/dietary; topical.	Preclinical: preserved RGCs; reduced oxidative stress and glial activation; increased NRF2 and normalized SOD2. Clinical: favourable VEP responses and less deterioration of RGC-layer/RNFL measurements.	Ex vivo and animal experiments; preliminary clinical evidence from combination treatment.	Acute injury models; limited oral absorption and formulation-dependent ocular delivery; vitamin E confounds attribution; sustained clinical neuroprotection unproven.
Pyruvate	DBA/2J mice and rat experimental glaucoma [57]; dietary supplementation in a mouse susceptibility model [59].	Observational association between circulating metabolites and glaucoma prevalence [59]. No standalone supplementation trial detailed; combination evidence below.	Preclinical: 500 mg/kg/day in the representative glaucoma experiments. Standalone clinical regimen not specified.	Preclinical: oral, drinking water.	Preclinical: restored retinal pyruvate; preserved RGC somas and axons; reduced ED1-positive microglial activation in rats. Human: higher energetic metabolites associated with lower glaucoma prevalence in individuals at high genetic risk.	Animal experiments; observational human association, without causal treatment evidence.	Association does not establish treatment benefit; optimal standalone human dose undefined; direct effects on specific inflammatory pathways in glaucoma unresolved.
Nicotinamide	DBA/2J mouse model of age-related glaucoma [60].	No standalone clinical trial detailed in the manuscript; nicotinamide + pyruvate trial evidence below.	Preclinical: 550 or 2000 mg/kg/day. Clinical combination regimens below.	Preclinical: oral, drinking water.	Preserved mitochondrial function and prevented molecular, functional and structural changes; at 2000 mg/kg/day, ~93% of treated eyes had no detectable RGC soma or axonal degeneration.	Animal experiments; human combination evidence does not isolate nicotinamide efficacy.	Glial responses not specifically assessed in the cited glaucoma model; sustained clinical neuroprotection unproven; high-dose hepatic safety and dose selection remain barriers [63].
Citicoline	Retinal cultures under excitotoxic/metabolic stress; optic nerve crush and chemically induced retinal injury models [73,74].	Small OAG electrophysiology studies; intermittent treatment followed for up to 8 years [88,89]. Oral/topical approaches also discussed [64,71,72].	Representative clinical regimen: 1000 mg/day IM in 60-day cycles, repeated intermittently. Preclinical and other-route doses not specified in the manuscript.	IM (clinical regimen); oral and topical routes also studied.	Preclinical: reduced apoptosis, preserved RGCs and neurites. Clinical: improved/stabilized PERG and VEP; partial deterioration during washout supports treatment-dependent neuroenhancement.	Cell and animal experiments, including non-glaucomatous models; limited clinical evidence based mainly on electrophysiological endpoints.	Small cohorts; heterogeneous routes and intermittent schedules; surrogate endpoints; no neuroinflammatory biomarkers; durable structural protection and true regeneration unproven.
Pyruvate + nicotinamide	Complementary mitochondrial-substrate and NAD^+^ rationale [32,49]; no combination-specific preclinical experiment detailed in the manuscript.	Phase II randomized, double-masked, placebo-controlled trial in treated OAG [61]; subsequent phase III trial design [62].	Phase II: nicotinamide 1000–3000 mg/day + pyruvate 1500–3000 mg/day, escalated. Phase III design: nicotinamide 3 g/day + calcium pyruvate 1 g/day.	Oral.	Phase II: more VF locations improved beyond test–retest variability; no serious adverse events during short follow-up. Phase III design evaluates longer-term VF and OCT outcomes.	Phase II randomized trial supporting short-term neuroenhancement; phase III design is not an efficacy result.	Short follow-up; progression benefit and synergy unproven; individual contributions unresolved; high-dose nicotinamide requires hepatic safety monitoring [63].
CoQ10 + citicoline	Mouse laser-induced ocular hypertension; retinal and central visual pathway analyses [45,46].	No combination-specific clinical study detailed in the manuscript.	CoQ10 200 mg/kg/day + citicoline 500 mg/kg/day, starting 15 days before ocular hypertension.	Oral.	Preserved Brn3a-positive RGCs; reduced retinal and central macroglial/microglial activation, including GFAP and P2RY12-related responses.	Animal experiments with combination treatment; clinical efficacy not established.	No monotherapy arms: individual effects and synergy cannot be determined; pretreatment limits extrapolation to established disease; human efficacy remains unproven.

## 5. Synergistic Strategies: Citicoline, Pyruvate, CoQ10 and Nicotinamide

In recent years, it has been proposed that combining various neuroprotective molecules may represent a more effective strategy than using individual therapeutic agents [32,90]. In particular, citicoline, pyruvate, CoQ10, and nicotinamide act on different aspects of neuronal energy metabolism and mitochondrial function [60].

These mechanisms provide a rationale for targeting complementary points in the mitochondrial–neuroinflammatory feedback loop [12,13,91]. Recent reviews also propose potential synergy between citicoline and coenzyme Q10 in retinal neurodegenerative diseases [92].

Nicotinamide-mediated NAD^+^ restoration provides a complementary component of this strategy [60].

The stage-dependent glial response is relevant to combination therapy [14,16]. The retinal glial findings for CoQ10 plus citicoline provide experimental support for this approach [46].

### Clinical Positioning: Priority Candidate Populations

The available evidence does not yet support routine disease-modifying use of these compounds in unselected patients with glaucoma. Nevertheless, a mechanism-based clinical framework identifies three populations in whom metabolic intervention is especially relevant for prospective evaluation and individualized consideration.

(1) Normal-tension glaucoma. In NTG, progressive RGC and optic-nerve injury occurs without IOP exceeding the statistically normal range. Because pressure elevation is not the dominant identifiable driver, mitochondrial, vascular, metabolic, oxidative, and neuroinflammatory vulnerability should be addressed from diagnosis rather than only after failure of pressure-lowering therapy. Metabolic neuroprotection may therefore be prioritized as an early, mechanism-directed strategy alongside individualized IOP management and assessment of ocular perfusion, while its use as established first-line pharmacotherapy awaits confirmatory clinical trials.
(2) Progression despite target IOP. Patients with documented structural or functional progression after achieving an individualized target IOP represent a second high-priority group. Continued RNFL loss, ganglion cell complex thinning, or reproducible visual-field deterioration suggests that pressure-independent mechanisms remain active. In this setting, metabolic intervention may provide a rational means of supporting dysfunctional but still viable RGCs, improving cellular resilience, and slowing further loss; however, progression should first be confirmed, and adherence, IOP fluctuation, vascular factors, and alternative diagnoses reassessed.
(3) Older patients and patients with systemic metabolic comorbidities. Aging is associated with declining NAD^+^ availability, impaired mitochondrial quality control, reduced antioxidant capacity, and lower neuronal metabolic reserve. Diabetes, insulin resistance, dyslipidemia, and other systemic metabolic disorders may further increase oxidative stress, microvascular dysfunction, and mitochondrial burden. In parallel, emerging ocular ageing biomarkers may provide additional tools for biological-age assessment and risk stratification. Retinal and OCT-derived parameters, including AI-based estimates of ocular age, have shown substantial ability to capture age-related phenotypic variation and may provide information relevant to disease-risk stratification [93]. The potential of retinal imaging to capture systemic metabolic information is further supported by recent artificial-intelligence frameworks capable of identifying conditions such as type 2 diabetes, hypertension, and hyperlipidemia from color fundus photographs [94]. Although these imaging approaches have not been validated for predicting treatment response in glaucoma, they illustrate the potential role of imaging-derived phenotypes in future multimodal metabolic and vascular risk stratification. These patients may therefore have less capacity to tolerate glaucomatous stress and may be particularly suitable for biomarker-guided metabolic strategies, with attention to comedications, renal and hepatic function, nutritional status, and compound-specific safety.

This stratification is intended to guide clinical-trial enrichment and individualized risk assessment rather than to imply proven efficacy. Future studies should prospectively test whether NTG phenotype, progression at target IOP, age, systemic metabolic disease, or biomarkers of mitochondrial and neuroimmune dysfunction predict treatment response.

## 6. Conclusions

Glaucoma is best framed as a complex neurodegenerative disease in which IOP-related injury is one of several interacting stress amplifiers. Mitochondrial dysfunction, oxidative stress, metabolic impairment, vascular dysregulation, excitotoxicity, and chronic neuroinflammation can both interact with pressure-related load and sustain degeneration when IOP is statistically normal or has been lowered to target. This framework is especially relevant to NTG and to patients who continue to progress despite adequate pressure control. Coenzyme Q10, nicotinamide, pyruvate, and citicoline act on distinct but interconnected components of these pathways, providing a strong mechanistic rationale for investigating them as core components of a broader, mechanism-based neuroprotective strategy rather than only as ancillary responses to elevated IOP.

The complementary actions of these compounds provide a rationale for targeting the mitochondrial–neuroinflammatory feedback loop. Their effects on neuroinflammation should be interpreted predominantly as secondary immunometabolic consequences of improved cellular homeostasis rather than conventional anti-inflammatory actions. This model is mechanistically compelling for NTG and progression despite target IOP, but direct demonstration that these agents interrupt the complete loop in human glaucoma is still lacking.

Experimental evidence consistently supports the ability of these compounds to preserve RGCs, improve mitochondrial resilience, reduce oxidative injury, and, in selected models, attenuate glial activation. However, direct evidence that any of these agents suppresses specific neuroinflammatory pathways in human glaucoma remains limited. Moreover, several of the most relevant preclinical and clinical studies evaluated combined formulations, including citicoline with CoQ10, CoQ10 with vitamin E, and nicotinamide with pyruvate. Consequently, the individual contribution of each compound and the presence of additive or synergistic effects remain incompletely defined.

The available clinical literature is also limited by relatively small sample sizes, heterogeneous study designs, differences in formulation, dosage, route of administration, and treatment duration, and the frequent use of electrophysiological or other surrogate endpoints. Short-term improvements in visual-field sensitivity, pattern electroretinography, or visual evoked potentials may indicate neuroenhancement of dysfunctional but viable neurons, but they do not necessarily demonstrate long-term neuroprotection, structural preservation, or true neuroregeneration. At present, no compound reviewed here has definitive clinical evidence demonstrating sustained disease modification in glaucoma.

Future studies should identify the patients most likely to benefit from metabolic neuroprotection, explicitly including NTG and pressure-resistant or target-IOP progressors, and determine whether baseline metabolic, mitochondrial, vascular, inflammatory, or genetic profiles predict treatment response. Adequately powered randomized clinical trials should stratify participants by glaucoma phenotype and achieved IOP, use standardized formulations and appropriate safety monitoring, provide sufficiently long follow-up, and include clinically meaningful structural and functional endpoints. Integrating VF testing and OCT with biomarkers of NAD^+^ metabolism, mitochondrial dysfunction, oxidative stress, glial activation, ocular perfusion, and neuroimmune dysregulation may establish whether biological target engagement translates into preservation of RGCs and visual function independently of further IOP reduction.

Combination strategies are particularly attractive because glaucoma involves several mutually reinforcing pathological mechanisms. Future studies should include appropriate monotherapy and combination-treatment arms to distinguish the specific contribution of each molecule and determine whether combined administration provides true additive or synergistic benefit. Coenzyme Q10, nicotinamide, pyruvate, and citicoline should therefore be considered investigational, pathway-directed interventions with particular clinical relevance to NTG and to glaucoma that progresses despite target IOP. They do not replace effective pressure control where pressure is modifiable, but neither should they be conceptualized solely as secondary adjuncts to IOP lowering. Instead, they represent candidate components of a comprehensive treatment paradigm that addresses fundamental metabolic, mitochondrial, oxidative, excitotoxic, and neuroinflammatory mechanisms not fully controlled by pressure reduction. Definitive positioning as disease-modifying therapy will require robust evidence of sustained structural and functional benefit.

## Figures and Tables

**Figure 1:**
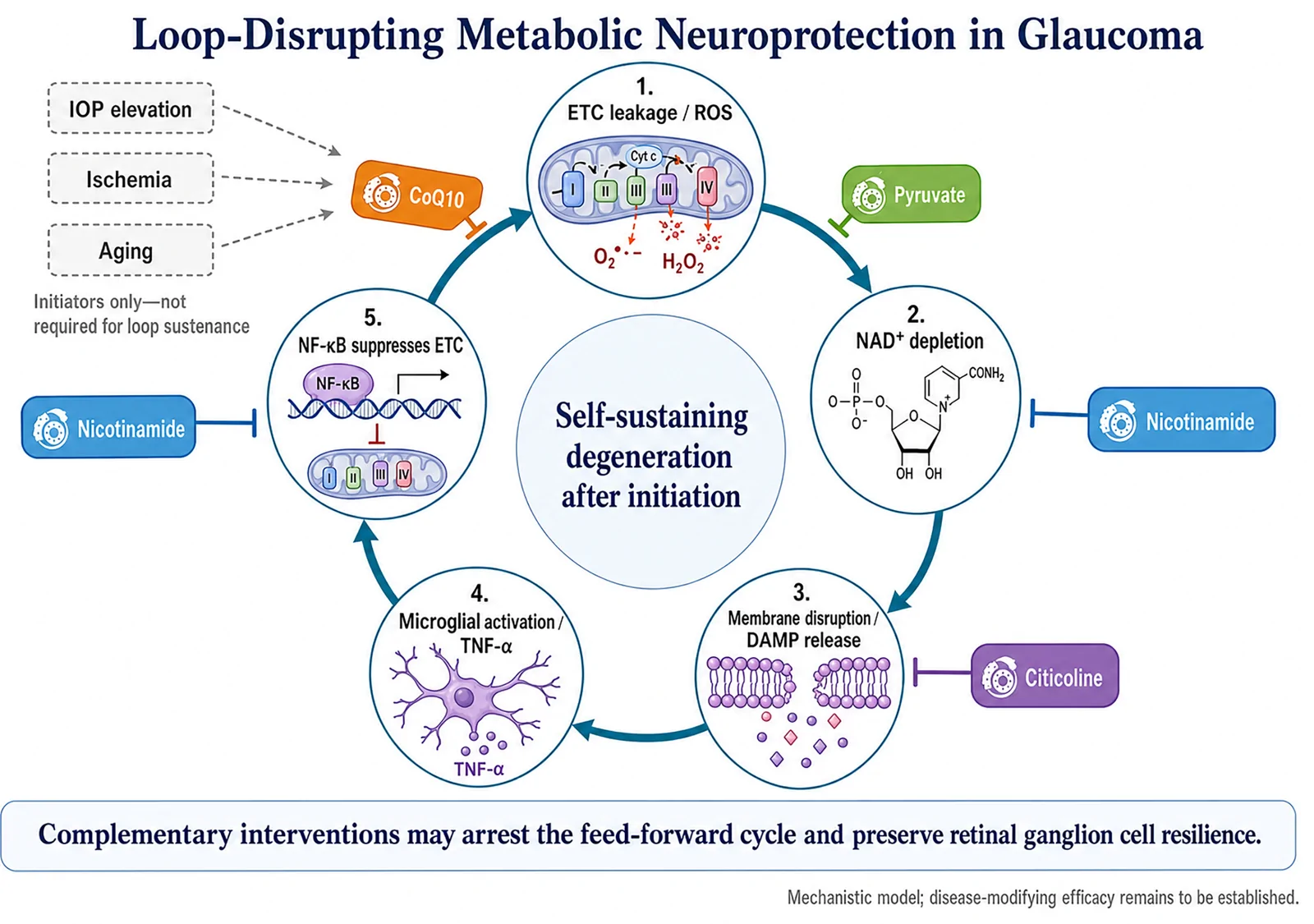
Proposed self-sustaining mitochondrial–neuroinflammatory loop and sites of metabolic intervention in glaucoma. External stressors—including intraocular pressure elevation, ischemia, and aging—may initiate mitochondrial electron transport chain dysfunction and reactive oxygen species production. Subsequent nicotinamide adenine dinucleotide depletion, membrane disruption, damage-associated molecular pattern release, microglial activation, and tumor necrosis factor-α/nuclear factor-κB signaling may further suppress mitochondrial respiration, establishing an IOP-independent feed-forward cycle. Coenzyme Q10, nicotinamide, pyruvate, and citicoline are positioned as candidate “loop-disrupting” interventions acting at complementary metabolic, mitochondrial, redox, and membrane-associated nodes. This mechanistic model remains to be validated clinically. Abbreviations: CoQ10, coenzyme Q10; DAMPs, damage-associated molecular patterns; ETC, electron transport chain; IOP, intraocular pressure; NAD^+^, oxidized nicotinamide adenine dinucleotide; NF-κB, nuclear factor kappa-light-chain-enhancer of activated B cells; ROS, reactive oxygen species; TNF-α, tumor necrosis factor alpha.

## Data Availability

Data sharing is not applicable to this article as no new data were created or analyzed in this study.
